# Animal Models for the Investigation of P2X7 Receptors

**DOI:** 10.3390/ijms24098225

**Published:** 2023-05-04

**Authors:** Ronald Sluyter, Sahil Adriouch, Stephen J. Fuller, Annette Nicke, Reece A. Sophocleous, Debbie Watson

**Affiliations:** 1 Molecular Horizons and School of Chemistry and Molecular Bioscience, University of Wollongong, Wollongong, NSW 2522, Australia; reeces@uow.edu.au (R.A.S.); dwatson@uow.edu.au (D.W.); 2 Illawarra Health and Medical Research Institute, Wollongong, NSW 2522, Australia; 3 UniRouen, INSERM, U1234, Pathophysiology, Autoimmunity, and Immunotherapy, (PANTHER), Univ Rouen Normandie, University of Rouen, F-76000 Rouen, France; sahil.adriouch@univ-rouen.fr; 4 Sydney Medical School Nepean, Faculty of Medicine and Health, The University of Sydney, Nepean Hospital, Kingswood, NSW 2750, Australia; stephen.fuller@sydney.edu.au; 5 Walther Straub Institute of Pharmacology and Toxicology, Faculty of Medicine, LMU Munich, 80336 Munich, Germany; annette.nicke@lrz.uni-muenchen.de

**Keywords:** P2X7 receptor, *P2RX7* gene, *P2rx7* gene, purinergic receptor, isoform, polymorphism, inflammation, immunity, transgenic animal, xenograft

## Abstract

The P2X7 receptor is a trimeric ligand-gated cation channel activated by extracellular adenosine 5′-triphosphate. The study of animals has greatly advanced the investigation of P2X7 and helped to establish the numerous physiological and pathophysiological roles of this receptor in human health and disease. Following a short overview of the P2X7 distribution, roles and functional properties, this article discusses how animal models have contributed to the generation of P2X7-specific antibodies and nanobodies (including biologics), recombinant receptors and radioligands to study P2X7 as well as to the pharmacokinetic testing of P2X7 antagonists. This article then outlines how mouse and rat models have been used to study P2X7. These sections include discussions on preclinical disease models, polymorphic P2X7 variants, P2X7 knockout mice (including bone marrow chimeras and conditional knockouts), P2X7 reporter mice, humanized P2X7 mice and P2X7 knockout rats. Finally, this article reviews the limited number of studies involving guinea pigs, rabbits, monkeys (rhesus macaques), dogs, cats, zebrafish, and other fish species (seabream, ayu sweetfish, rainbow trout and Japanese flounder) to study P2X7.

## 1. Introduction

Purinergic signaling comprises a network of P1, P2X and P2Y receptors activated by extracellular adenosine, adenosine 5′-triphosphate (ATP) and other nucleotides and regulated by the metabolism, release and uptake of nucleotides and nucleosides [1]. P2X receptors are trimeric ligand-gated cation channels formed by the homomeric or heteromeric assembly of P2X subunits (P2X1–P2X7) and are activated by extracellular ATP [2]. Of the P2X receptor members, the P2X7 receptor (P2X7) has been the most widely studied, which is largely due to its presence in leukocytes and lymphoid organs as well as its various roles in inflammation and immunity and related disorders including infectious diseases [3]. Various physiological and pathophysiological roles of P2X7 have also been found in many organs and tissues including those of the bone [4], cardiovascular system [5,6], eye [7], exocrine system [8], gastrointestinal tract [9], kidney [10], liver [11], lung [12], nervous system [13,14], skeletal muscle [15] and skin [16], and it has further been involved in cancer [17,18], pain [19] hematopoiesis [20], and metabolism [21]. This broad range of roles reflects the wide cellular and tissue distribution of P2X7, which might partly reflect its dominant presence in leukocytes compared to other cell types [22], while its presence in neurons remains debated [23,24].

P2X7 typically assembles and functions as a homomeric receptor [25,26,27], but heteromeric receptors with P2X4 subunits have been observed [28]. Both subunits show the highest homology among the P2X family members, and their genes (*P2RX7* and *P2RX4*, respectively) are closely located on the same chromosome in humans and other animals [29]. In humans and other mammals, the *P2RX7* gene is comprised of 13 exons [22]. P2X7 isoforms are reported in various animals, but these have only been studied in humans [30,31,32,33], mice [34,35] and rats [35], with P2X7A and P2X7a considered to represent the canonical P2X7 type in humans and rodents, respectively.

In addition to extracellular ATP, P2X7 can be activated by the experimentally used agonist 2′(3′)-O-(4-benzoylbenzoyl) ATP (BzATP) and by the partial agonists adenosine-5′-O-(3-thio) triphosphate and 2-methylthio-ATP [36,37] (Table 1). In some species, including mice [38] and rats [39], P2X7 can be irreversibly activated by another endogenous ligand, nicotinamide adenosine dinucleotide (NAD^+^). This involves the adenosine 5′-diphosphate (ADP)-ribosylation of the Arg125 residue of P2X7 by ecto-ADP-ribosyl transferase 2 (ARTC2) [40]. However, this process is absent in species such as humans and other primates due to the lack of functional ARTC2 [41]. The activation of P2X7 by NAD^+^ is further complicated by occurring in an isoform-specific manner, as shown by the activation of the murine P2X7 variant, P2X7k, via ADP-ribosylation but not of the widely distributed P2X7a [42,43].

P2X7 activation typically results in an influx of Ca^2+^ and Na^+^ and efflux of K^+^ as well as the formation of an incompletely defined pore allowing the flux of organic ions, including choline, spermidine, and ATP [45], as well as fluorescent dyes, which are often used to detect and quantify P2X7 activity [46]. An emerging role of P2X7 pore formation is the uptake of cyclic guanosine 5′-monophosphate, released from stressed or dying cells, to stimulate STING-dependent interferon-β production in macrophages [47]. P2X7 activation also results in an array of other downstream events [48], including activation of the NLRP3 inflammasome and caspase-1, gasdermin D pore formation, and consequent activation and release of interleukin (IL)-1β and IL-18 pro-inflammatory cytokines [49], cell surface receptor shedding [50], autophagy, cell proliferation and cell death [51]. In addition, it also leads to the intracellular destruction of pathogens [52]. In the absence of its activation by extracellular ATP, P2X7 can further function as a phagocytic receptor capable of binding and facilitating the phagocytosis of prokaryotic and eukaryotic cells [53].

While the functional and pharmacological properties of P2X7 receptors from different species vary significantly [54], current understanding of the human P2X7 has been greatly aided by the study of animals. This article will discuss how animals in general have contributed to the generation of antibodies and nanobodies against P2X7 (used as tools as well as biologics), the cloning or generation of recombinant forms of P2X7, the pharmacokinetic characterization of P2X7 antagonists, and the development of P2X7 radioligands as diagnostic tools. This article will then review the various animal models used to investigate P2X7, which have helped to establish the roles of this receptor in human health and disease. Articles were identified using relevant search terms in PubMed (https://pubmed.ncbi.nlm.nih.gov/) (last accessed on 31 March 2023) with additional articles identified through Google Scholar (https://scholar.google.com/) (last accessed on 31 March 2023) and manual searching of articles.

## 2. Uses of Animals to Study P2X7

### 2.1. Antibodies and Nanobodies

As in the case of other receptors and potential drug targets, the study of animals has greatly advanced the investigation of P2X7. Most commonly, this has been achieved through the direct molecular, biochemical, immunohistochemical, and functional analyses of cells, tissues and organs from animals, particularly mice and rats [55]. Related to this is the indirect use of animals to generate antibodies or nanobodies against human and rodent P2X7 as tools for P2X7 detection and in some instances even with inhibitory or potentiating properties and potential use as biologics [56]. Classical procedures included the use of rabbits to raise anti-P2X7 polyclonal antibodies [57,58] as well as the use of rodents to produce murine anti-human P2X7 monoclonal antibodies (mAbs), such as clones L4 [59] and 4B3A4 [60] and rat anti-murine P2X7 mAbs, such as clones Hano43 [57] and 1F11 [61], the latter of which can inhibit murine P2X7. Clone L4, which detects an extracellular epitope and has inhibitory properties [59], has been used in a variety of applications [62]. Together, this has prompted the commercial availability of a wide array of anti-P2X7 polyclonal antibodies (e.g., Cat. No. APR-004 and APR-008, Alomone Labs, Jerusalem, Israel) and some anti-P2X7 mAbs (e.g., Cat. No. 148702, BioLegend, San Diego, CA, USA; Cat. No. ALX-802-027, Enzo Life Sciences, Farmingdale, NY, USA). Antibodies have also allowed the development of an immunoassay (Cusabio, Houston, TX, USA) to quantify circulating, soluble P2X7 [63], which may have diagnostic potential in sepsis [64], *Mycoplasma pneumoniae* pneumonia [65], acute myocardial infarction [66], COVID-19 [67], and epilepsy [68], but not schizophrenia [69].

Recent biotechnological approaches led to the development of anti-human (Dano1) and anti-murine P2X7 (13A7 and 14D5) nanobodies from lamas [70]. Such nanobodies have proven useful in the identification of P2X7-expressing cells in tissues and organs from rodents [71,72] and humans [73] and as potential biologics to attenuate disease, as already shown in mouse models of inflammatory [70] and neurological [74] disorders as well as cancer [75]. Moreover, anti-P2X7 nanobodies have been used to engineer capsid-modified adeno-associated viral (AAV) vectors [76], which enables the selective transduction of P2X7-expressing cells. Another interesting development is the generation and use of AAV vectors coding for anti-P2X7 nanobodies to provide a safe, stable, and long-lasting nanobody-mediated in vivo blockade of P2X7 following a single AAV vector injection [77]. The latter procedure was shown to be capable of inhibiting P2X7 at the surface of circulating immune cells but also in brain microglia [78], and it was used to demonstrate the role of P2X7 in tumor growth [79] in mice models. This methodological approach represents an alternative to P2X7 knockout or knockdown procedures but also allows for the long-term effects of biologics targeting P2X7 in vivo to be studied.

### 2.2. Recombinant Receptors

Since the initial cloning of P2X7 (originally termed P2Z) from the rat [37] and human [80], P2X7 from diverse animal species were cloned (Table 2) and advanced the investigation of P2X7 by structural, functional, and pharmacological studies [81,82]. For example, a milestone in P2X7 research was the determination of the first P2X7 crystal structure, which was achieved by screening truncated P2X7 versions from 10 different mammalian species. The successfully solved structure of P2X7 from the Giant Panda identified an allosteric drug binding pocket, to which most P2X7 antagonists bind, and it helped to reveal their mechanism of action [83]. More recently, cryo-electron microscopy of a recombinant rat P2X7 yielded the first full-length structure of P2X7, revealing for the first time its cytoplasmic features such as a palmitoylated “C-Cys anchor domain”, which prevents desensitization and a globular “ballast domain” containing a dinuclear Zn^+^ complex and a high-affinity guanosine nucleotide binding site [84], which may serve to stabilize the trimeric complex [85] independently from the extracellular ATP binding domain [86].

### 2.3. Pharmacokinetic Studies and Radioligands

Animals have served as models to evaluate the pharmacokinetic properties and safety of several P2X7 antagonists. For example, CE-224,535 showed excellent pharmacokinetics and safety in rats, dogs, and monkeys [87], thereby paving the way for a first Phase IIA trial conducted by Pfizer in people with rheumatoid arthritis, where this drug was also observed to be well tolerated and safe [88]. Together with the absence of a noxious phenotype in *P2rx7* gene knockout (*P2rx7* KO) animals (Section 3.3), this represented a critical step for the use of P2X7 inhibitors in future studies and disease settings. However, for yet unknown reasons, the therapeutic value of the compound in humans remained behind the expectations despite promising animal data. Different reasons, such as splice variants or single nucleotide polymorphisms (SNPs) with altered functionality, agonist specificities, and/or pharmacokinetic properties might account for this.

Since P2X7 is considered a drug target for different diseases of the central nervous system, including neurodegenerative and neuropsychiatric disorders and neuropathic pain, the development of centrally available antagonists came into focus [89]. More recently, the potential utility of radiolabeled P2X7 ligands as diagnostic imaging agents in vivo, particularly within the central nervous system, was shown [90]. Here, nonhuman primates have, for example, been used for the preclinical evaluation of [^11^C]GSK1482160 [91] and [^18^F]JNJ-644133739 [92]. In addition, studies using these or other radiolabeled P2X7 ligands in rodent models further highlight their potential as markers of neuroinflammation [93], inflammation in cancer [94] or atherosclerotic lesions [95].

**Table 2:** Recombinant forms of P2X7.

Species	Length (Amino Acid Residues) ^1^	Identity to Human P2X7 (%) ^1^	ATP EC_50_ (μM) ^2,3^	BzATP EC_50_ (μM) ^3^	Reference
Human	595	100	779 ^4^	52 ^4^	[80]
Rhesus macaque	595	97	802	58	[96]
Dog	595	86	3162	501	[97]
Giant panda	595	85	122	N.R.	[83]
Mouse	595	81	734	90	[98]
Rat	595	80	115	7	[37]
Guinea pig	594	77	603	>100	[99]
Seabream	576	46	1840	130	[100]
Japanese flounder	580	46	790	743	[101]
African clawed frog	553	45	2600	139	[102]
Zebrafish	596	42	109 ^5^	19 ^5^	[103]

^1^ Values from [22]. ^2^ Abbreviations: ATP, adenosine 5’-triphosphate; BzATP, 2′(3′)-O-(4-benzoylbenzoyl) ATP; EC_50_, half-maximal effective concentration; N.R., not reported. ^3^ EC_50_ values determined from electrophysiology recordings, except guinea pig P2X7 determined from dye uptake measurements. ^4^ EC_50_ values from [98]. ^5^ EC_50_ values from [100].

### 2.4. Physiology and Pathophysiology Studies

Physiology and pathophysiology are complex processes not easily replicated by the study of cells, tissues or organs ex vivo, or through bioengineering or computational techniques. In the absence of sufficient sources of human tissues, organs or models, the use of animal models remains necessary to study the role of P2X7 in health and disease, for the future benefit of both people and animals through the development of new knowledge, and potential biomarkers, medicines, and therapies. However, research with such models, including animal care, requires the application of ethical, humane, and responsible principles and practices including but not limited to institutional approval and compliance and consideration of the principles of replacement, reduction and refinement (the 3Rs) [104], ARRIVE Guidelines [105] and other field specific guidelines. Examples of the latter are those provided for studying drugs for the treatment of amyotrophic lateral sclerosis in rodents [106,107]. Despite their value, field-specific guidelines may contain limitations [108] and therefore should be carefully considered in the broader context of other knowledge and resources available. Given the value of animal models, the remainder of this article will provide an overview of mouse and rat models used to study P2X7. Finally, this article will describe the limited number of studies involving guinea pigs, rabbits, rhesus macaques, dogs, cats, zebrafish, and other fish species that have investigated P2X7.

## 3. Mice

### 3.1. Preclinical Mouse Models

Mice (*Mus musculus*) have been extensively used to study the role of P2X7 in a wide range of preclinical disease models and to ascertain the therapeutic efficacy of targeting P2X7 with either small molecule inhibitors, biologics, or gene knockdown strategies [54,109]. While these studies have greatly advanced the understanding of the physiological and pathophysiological roles of P2X7, certain caveats need to be considered. Although it is beyond the scope of this article to review every study to date, the comparison of a small number of preclinical studies reveals several salient points for consideration in interpreting past studies and for future investigations of P2X7 in mice, which can be applied to other animal models.

#### 3.1.1. Selection of Antagonists

A large proportion of preclinical mouse studies to date have used the P2X7 antagonist Brilliant Blue G (BBG) [110], which is largely due to its wide availability, ease of use and extremely low cost compared to other P2X7 antagonists [54]. In addition, BBG is very similar to Brilliant Blue FCF, which is an approved food colorant [111]. As such, BBG remains a valuable drug for preliminary proof-of-concept studies, but unspecific effects need to be considered. For example, BBG can bind to and inhibit various proteins [112] including other P2X receptors, albeit with lower efficacy than P2X7 [110,113]. In addition, BBG can inhibit the ATP-release channel pannexin-1 [114] and neuronal voltage-gated Na^+^ channels [115], and it can also impair prion activity [116] and amyloid fibril formation [117] and conversely promote α-synuclein aggregation [118]. Thus, effects on P2X7 need to be confirmed using more specific compounds. The limited ability of BBG to cross the blood–brain barrier at high concentrations [119] and its accumulation in the peripheral organs of mice [120] further reduce its suitability. Importantly, BBG, but not the specific P2X7 antagonist JNJ-47965567 [121], failed to impair P2X7-mediated release of IL-1β in a human whole blood assay [122], raising uncertainty as to whether BBG can block P2X7 on circulating blood cells or cells in other extracellular milieus, and questioning its efficacy in various disorders in vivo. Finally, significant differences in species specificity have been observed for BBG and other P2X7 antagonists [54].

#### 3.1.2. Dosing of Antagonists

In many of the reported preclinical mouse models, the antagonist dosage regimens appear to be based on empirical evidence rather than pharmacokinetic data. For example, BBG was originally used in rats, where a single intraperitoneal (i.p.) injection of 100 mg/kg but not 40 mg/kg BBG reduced lipopolysaccharide-induced fever [123]. In a later study, 10 mg/kg BBG delivered from pellets attenuated experimental autoimmune encephalitis (a model of human multiple sclerosis) in rats [124]. The first reported study using BBG in mice demonstrated that 45.5 mg/kg BBG injected i.p. every second day attenuated Huntington’s Disease [125]. While these studies provided an important basis for further P2X7 research, the BBG dosage regimen was not explained. Subsequently, a dose of approximately 50 mg/kg BBG i.p. every second day or three times a week became widely adopted and shown, for example, to reduce amyotrophic lateral sclerosis in Glu93Ala superoxidase dismutase 1 mice [126,127,128]. In one of these studies [126], 250 mg/kg BBG three times per week was shown to further improve health and motor coordination, suggesting that 50 mg/kg BBG may be sub-optimal and highlighting the need for the optimization of dosing regimens. Likewise, in a humanized mouse model (see Section 3.5), daily injections of 50 mg/kg BBG (Days 0–10) were superior in reducing graft-versus-host disease (GVHD) [129], a frequent complication of allogenic hemopoietic stem cell transplantation [130], than 50 mg/kg BBG every second day (Days 0–8) [131]. Interestingly, daily injections of the non-selective P2X7 antagonist pyridoxalphosphate-6-axophenyl-2′-4′-disulfonic acid [132] at 300 mg/kg (Days 0–10) only partly reduced GVHD [129]. Furthermore, daily injections of the P2X7 antagonist AZ101606120 [133] at 2 mg/kg (Days 0–10) did not show an effect [134].

To enable comparability, it is important to optimize and standardize procedures, and the field may benefit from an international consensus and recommendations regarding these matters. The development of anti-P2X7 nanobodies with extended circulating half-lives [70] and the generation of AAV vectors encoding these nanobodies, capable of sustained, safe nanobody production over 100 days [77] and central nervous system penetration [78], afford alternative strategies to investigate P2X7 in mouse models of disease as well as new potential therapies in people and other animals. Finally, the use of agonists and positive modulators as tools in P2X7 research should also be mentioned. For example, the small molecule P2X7 activator HEI3090 in combination with immune checkpoint blockade was recently shown to enhance anti-tumor immune responses in mice [135]. Interestingly, a potentiating anti-P2X7 nanobody (14D5), which can augment inflammatory disorders in mice, has also been developed [70]. Such biologics provide opportunities to treat pathophysiological conditions in which P2X7 activity is insufficient or needs to be augmented.

### 3.2. Polymorphic P2X7 Variants in Mice

**Figure 1:**
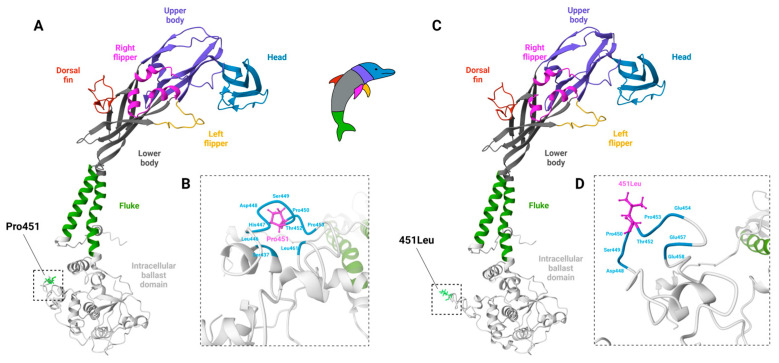
Molecular models of the mouse P2X7 subunits with amino acid residue Pro451 or 451Leu. (**A**–**D**) The full-length mouse P2X7 subunit (NCBI) was modeled with the I-TASSER protein structure prediction suite [142,143] using the cryo-electron microscopy structure of rat P2X7, PDB ID: 6u9v [84] as a template. Sections of the P2X7 subunits are colored as depicted by the dolphin-like structure (inset) of the P2X4 subunit [144], including the head (blue), upper body (violet), right (magenta) and left (yellow) flippers, dorsal fin (red), lower body (gray), transmembrane domain-spanning fluke (green), and the intracellular ballast domain (not shown on inset; white). Boxed areas indicate the region containing the Pro451Leu SNP. (**A**) Modeling of P2X7-Pro451 returned a C-score of 0.25 with an estimated TM-score of 0.75 ± 0.11 and root mean square deviation of 7.1 ± 4.2 Å. (**B**) Close-up images of boxed areas, with Pro451 shown as ball and stick structures (magenta). Amino acid residues within 5 Å of the residue of interest are also labeled (cyan). (**C**) Modeling of P2X7-451Leu returned a C-score of 0.20 with an estimated TM-score of 0.74 ± 0.11 and root mean square deviation of 7.2 ± 4.2 Å. (**D**) Close-up images of boxed areas, with Leu451 shown as ball and stick structures (magenta). Amino acid residues within 5 Å of the residue of interest are also labeled (cyan). Images were produced using Mol* [145]. Figure created in BioRender.com with permission.

When using mice to study P2X7, it needs to be considered that SNPs with altered functionality may exist, including a loss-of-function SNP of P2X7 present in several commonly used mouse strains (Table 3). This SNP encodes for a proline to leucine substitution at residue 451 in the cytoplasmic C-terminus of P2X7 (Figure 1), impairing receptor function in several assays [136]. Compared to murine P2X7-Pro451, the heterologous expression of murine P2X7-451Leu in human embryonic kidney (HEK)-293 cells results in reduced agonist-induced Ca^2+^ fluxes and dye uptake as well as reduced ATP-induced phosphatidylserine exposure [136,137]. Furthermore, when expressed in HEK-293 cells, the Pro451Leu SNP impaired agonist-induced dye uptake in the context of the P2X7a variant but not in the context of the P2X7k [43], which is 8-fold more sensitive to agonist and displays slower deactivation kinetics [35]. It is important to note that agonist-induced Ca^2+^ fluxes and dye uptake were similar for Pro451 and 451Leu variants if they were transfected in human 1321N1 cells [138]. Furthermore, the same mutation in recombinant human P2X7 does not impair dye uptake in HEK-293 cells [139]. Thus, the region around residue 451 may mediate a cell-type-specific interaction that may depend on differential intracellular signaling mechanisms. In line with this notion, this SNP is in the C-terminal tail of the receptor (Figure 1) in the globular “ballast domain”, which is believed to play an important role in the stability of the homotrimeric receptor as well as in intracellular signalization.

**Table 3:** Distribution of the Pro451Leu SNP in the *P2rx7* gene of commonly used mouse strains.

Pro451 ^1^	451Leu ^1^
129/J, 129S1, 129X1/SvJ, A/He, A/J, BALB/c, BALB/cAnNCrl, BALB/cByJ, BUB/Bn, New Zealand White (NZW), LG, LP, nonobese diabetic (NOD), MRL/Mp	AKR/J, B10.D2, C3H/HeJ, CALB/RkJ, C57BL/6, C57BL/6NCrl, C57BL/10, C57L/J, DBA/1, DBA/2, DDY/J, FVB/N, New Zealand Black (NZB), SJL/J, SM/J, SWR/J

^1^ Data obtained from [136,140,141].

Functional differences between Pro451 and 451Leu P2X7 variants are also observed in murine primary cells. Agonist-induced Ca^2+^ fluxes, phosphatidylserine exposure, CD62L shedding and cell death are lower in T cells from C57BL/6 or New Zealand Black mice (451Leu) than in T cells from BALB/c or New Zealand White mice (Pro451) [136,146]. Likewise, agonist-induced IL-1β release is reduced in splenocytes from New Zealand Black (451Leu) mice compared to splenocytes from New Zealand White (Pro451) mice [146]. Agonist-induced dye uptake is reduced in osteoclasts from C57BL/6 and DBA/2 (451Leu) mice compared to osteoclasts from BALB/c and 129X1/SvJ (Pro451) mice [141]. However, agonist-induced phospholipase D stimulation does not differ between thymocytes from BALB/c (Pro451) and C57BL/6 (451Leu) mice despite reduced agonist-induced cell death in the latter [147]. The differences in P2X7 activity appear to be partly due to reduced amounts of P2X7 since less P2X7 was observed on the cell surface of CD4^+^ and CD8^+^ T cells from C57BL/6 or FVB/N (451Leu) mice compared to T cells from 129 mice (Pro451) mice [148]. Notably, in this same study, it was found that the *P2rx7* Pro451 SNP is retained as a passenger mutation in C57BL/6 *P2rx4* gene knockout (*P2rx4* KO) mice that were generated using 129-derived stem cells. The proximity of the *P2rx4* and *P2rx7* genes makes the recombination during backcrossing very unlikely, and consequently, T cells of the *P2rx4* KO mice have higher P2X7 activity than C57BL/6 wild-type mice.

Remarkably, P2X7 451Leu-carrying mouse strains display also phenotypic differences, including reduced pain sensitivity [140], impaired glucose homeostasis (impaired glucose tolerance and insulin responsiveness) [149], and reduced bone strength [141]. Furthermore, this SNP was shown to diminish the differences in bone phenotype between *P2rx7* KO and wild-type mice in 451Leu and Pro451 genetic backgrounds [150], and the possibility was raised that the P2X7 451Leu passenger mutation could partly account for the bone phenotype in *P2rx4* KO mice [151]. Conversely, studies of *P2rx7* KO mice on both backgrounds revealed a limited but similar effect of the SNP on inflammatory and thermogenic P2X7 functions in white and brown adipocytes [152].

### 3.3. P2rx7 Gene Knockout Mouse Models

The first reported genetically modified P2X7 mouse models were conventional *P2rx7* KO mice (Table 4). The first such model was developed by GlaxoSmithKline and originally used for in vitro studies, demonstrating a non-essential role for P2X7 in the generation of nitric oxide formation in macrophages [153]. A detailed description of the generation of this *P2rx7* KO mouse (by LacZ-neomycin cassette insertion and deletion of exon 1, encoding the N-terminus and part of the first transmembrane domain), however, was only reported in two subsequent studies [154,155]. One of these studies established a role for P2X7 in chronic inflammatory and neuropathic pain in vivo [154], while the other study compared this and the Pfizer *P2rx7* KO strain and different anti-P2X7 antibodies to show the absence of P2X7 in hippocampal neurons [155]. The second reported *P2rx7* KO mouse was developed by Pfizer (by neomycin cassette insertion and deletion of exon 13, encoding the intracellular C-terminus) [156] and subsequently used to demonstrate a role for P2X7 in inflammatory arthritis [157]. A third *P2rx7* KO mouse, developed by Lexicon Genetics for Abbott Laboratories, revealed a role for P2X7 in depression [158]. A fourth *P2rx7* KO mouse, developed at Shanghai University using CRISPR/Cas9 technology, revealed that P2X7 protects from viral infection [159] and that P2X7 contributes to depression as mice age [160]. In addition, a partial *P2rx7* knockdown mouse was developed by the German Research Center for Environmental Health using short hairpin technology, which reduced *P2rx7* mRNA expression in the brain by 88% [161], but further characterization was not reported and to the best of our knowledge, this mouse model has not been studied further. In addition, conditional *P2rx7* KO mice have been generated using the Cre/loxP system and are further described below. Regarding the constitutive *P2rx7* KO mice, the Glaxo and in particular the Pfizer mice have been used in an estimated 400 original research articles to date. Some of these studies include the crossing of *P2rx7* KO mice with spontaneous disease models. Paradoxically, some of these studies revealed unexpected results compared to prior studies with P2X7 antagonists or inducible disease models in *P2rx7* KO mice, such as the enhancement of autoimmune arthritis in K/BxN mice [162].

In general, *P2rx7* KO mice display no gross developmental defects, although several phenotypic alterations have been reported in Pfizer *P2rx7* KO mice, which is arguably the most studied *P2rx7* KO mouse strain. These alterations include impaired bone development [166], reduced spatial memory [167], reduced obsessive behavior and aggression [168], increased rod and cone pathway post-photoreceptor responses [169], various protein alterations in the corneal stroma [170], features of early age-related molecular degeneration [171], decreased pancreatic stellate cells [172], increased body weight and altered fat distribution in older mice [173], increased triglycerides and cholesterol concentrations with impaired glucose homeostasis and evidence of hepatic steatosis [174], and decreased fatty acid oxidation and whole body energy expenditure [175]. Fewer phenotypic alterations have been reported in the other *P2rx7* KO mouse strains. Increased numbers of demyelinated axons of sciatic nerves and Remake bundles are present in GlaxoSmithKline *P2rx7* KO mice [176]. Reduced olfactory function has been observed in Shanghai University *P2rx7* KO mice with increased olfactory function in older mice [177]. Finally, as recently noted by others [2], the phenotyping of over 8300 different knockout mice by the International Mouse Phenotyping Consortium (https://www.mousephenotype.org) is available, with this website currently reporting that *P2rx7*^tm1a(EUCOMM)Wtsi^ knockout mice display decreased circulating glycerol concentrations, while some *P2rx7*^tm1b(EUCOMM)Wtsi^ knockout mice display abnormal lens morphology and early cataract development.

Early studies reported that there were no differences in the proportions of mature leukocytes in Pfizer *P2rx7* KO mice compared to wild-type mice [156,157]. A recent, more detailed examination of these mice has extended these findings, reporting similar numbers of mature blood cells and hematopoietic stem and progenitor cells but increased megakaryocyte erythroid progenitors and B cell precursors in the bone marrow from *P2rx7* KO mice [178]. Other studies have reported increased numbers of lymph node regulatory T cells [179], γδ thymocytes [180] and Peyer’s patches follicular helper T (Tfh) cells [181] as well as reduced mesenteric lymph node γδ T cells [180]. The increase in Tfh cells in *P2rx7* KO mice was associated with increased germinal center reactions and IgA secretion but reduced IgM production [181]. These changes were associated with reduced mucosal colonization [181] but enrichment of *Lactobacillus* colonization [182], leading to altered glucose homeostasis and increased body weight [182,183]. Thus, given the wide roles of the gastrointestinal microbiome and possibly other microbiomes in health and disease [184], consideration of changes to this microbiome should be considered when studying P2X7 in *P2rx7* KO mice. Whether changes in the microbiome also occur with the prolonged use of P2X7 antagonists or biologics remains to be determined.

Despite the potential presence of truncated P2X7 variants lacking the C-terminal tail (that remain to be demonstrated at the functional level) [185], the Pfizer *P2rx7* KO mouse is the most widely used *P2rx7* KO mouse, as it was made freely available by the original inventors [156] and subsequently through the Jackson Laboratory (Bar Harbor, ME, USA). In addition, a splice variant displaying higher sensitivity to agonists, the latter discovered P2X7k (592 amino acid residues in length), escapes gene inactivation in the GlaxoSmithKline *P2rx7* KO mice [35] and made this mouse a questionable alternative. In this splice variant, the N-terminus and part of the first transmembrane domain are encoded by an alternative exon 1′. The variant appears to be the dominant form in lymphocytes and leads to enhanced P2X7 activity in CD4^+^ and CD8^+^ T cells compared to T cells from wild-type mice, but P2X7 is absent in macrophages and dendritic cells from these knockout mice [42,186,187]. Thus, findings obtained using *P2rx7* KO mice should be carefully evaluated with respect to the possible presence of truncated forms and to the confirmed absence of both P2X7a and P2X7k variants in the different cell types to avoid possible bias and misinterpretations.

A further consideration when studying and enumerating cells in *P2rx7* KO and wild-type mice is the possibility of selection bias during tissue dissociation. The release of both NAD^+^ and ATP and the subsequent activation of P2X7 on T cells during tissue dissociation has been documented [188]. Although the effects of ATP can largely be avoided by dissociating tissues at 4 °C, the ADP-ribosylation of P2X7 can still occur at low temperatures and lead to P2X7 activation with phenotypic and functional changes in T cells when samples are returned to 37 °C [188]. The injection of etheno-NAD or an ARTC2-blocking nanobody (s+16a) prior to sacrificing mice can prevent NAD^+^-induced P2X7 activation and subsequent changes in T cells [188] or the loss of regulatory T cells, natural killer T cells, and/or CD4^+^ and CD8^+^ memory T cells during tissue processing [189,190,191].

*P2rx7* KO mice were also used to generate chimeric mouse models, in which bone from *P2rx7* KO or wild-type mice is transplanted into wildtype or *P2rx7* KO mice, respectively (along with corresponding controls) to delineate the contribution of P2X7 on hematopoietic and non-hematopoietic cells to physiology or pathology. Such studies have helped to reveal a role for P2X7 on host antigen-presenting cells in GVHD development [192], T cells in the protection from experimental autoimmune encephalitis [193], or more broadly hematopoietic cells in allergic airway inflammation [194], lung inflammation and emphysema [195], acute respiratory distress syndrome [196], mood disorders [197], and tumor immunity [198]. Such studies also revealed a role of P2X7 on non-hematopoietic cells, such as gastrointestinal epithelial cells, in the recruitment of dendritic cells during *Toxoplasma* and *Trichinella* infection [199]. However, contrasting roles for P2X7 on hematopoietic cells [200] and parenchymal cells [201] in promoting renal ischemia–reperfusion injury were also reported. Likewise, two other studies have revealed that P2X7 protects from tuberculosis [202] but can also contribute to severe tuberculosis progression [203]. Finally, a role for P2X7 on both hematopoietic and blood vessel cells in tissue factor-dependent thrombosis [204] was demonstrated.

More recently, several conditional *P2rx7* KO mice have been generated using the Cre/loxP system. A mouse generated by targeted mutation is available from the European Mouse Mutant Archive *P2rx7*^tm1a(EUCOMM)Wtsi^ [205], which upon crossing with flippase expressing mice leads to the *P2rx7*^tm1c(EUCOMM)Wtsi^ or floxed *P2rx7^fl/fl^* mouse. This can be further crossed with different cre-driver lines to obtain cell type- or tissue-specific *P2rx7* KO mice. A comparison of *P2rx7^fl/fl^* mouse with CD4^+^ T (with CD4-cre)-, myeloid cells (with LySM-Cre)- or airway epithelial cell (with CCT-cre)-specific *P2rx7* KO mice revealed a role for CD4^+^ T cell and myeloid cell P2X7 in the development of acute respiratory distress syndrome [196]. T cell-specific *P2rx7* KO mice have also helped to establish an intrinsic role for P2X7 on Tfh cells in limiting systemic lupus erythematous development [206]. The use of this system has revealed a role for P2X7 in the generation of CD8^+^ tissue resident memory T cells following viral meningitis infection [207]. Oligodendrocyte (with CNP-Cre)- or microglia (with CX3CR1-Cre)-specific *P2rx7* KO mice helped to confirm the presence of P2X7 in these cell types [71]. By crossing *P2rx7^fl/fl^* with LySM-Cre mice, it was shown that the small molecule P2X7 activator HEI3090 acts on macrophages to promote P2X7-mediated IL-18 production [135]. While the Cre/loxP system can prevent potential compensatory effects and help to determine cell-type or tissue-specific effects, careful characterization of the cre lines and respective *P2rx7* KO mice is required as incomplete or unexpected recombination can occur, such as during development [208,209].

Recently, a floxed *P2rx7* knockin–knockout mouse model (*P2rx7^KiKo^* mice) was described, in which the N-termini of the P2X7a and P2X7k variants were tagged with Flag-HA-AU1 and Flag-HA-IRS tags, respectively. Unexpectedly, the muscular P2X7 protein in this mouse was already ablated in the absence of cre recombinase. Nevertheless, this mouse revealed a role for macrophage but not muscle P2X7 in preventing dystrophic mineralization in Duchenne muscular dystrophy [210].

### 3.4. P2X7 Reporter Mouse Models

So far, two transgenic P2X7 mice have been generated and studied to determine P2X7 expression and distribution. The first, Tg(P2rx7-EGFP)FY174Gsat, was made available by the U.S. National Institute of Health Mutant Mouse Regional Resource Centers and subsequently the GENSAT Project. In this mouse, a soluble enhanced green fluorescent protein (sEGFP) is synthesized under the control of a bacterial artificial chromosome (BAC)-derived transgenic *P2rx7* gene promotor (www.gensat.org/about.jsp), which is named here the sEGFP reporter mouse. The use of this mouse was first reported by Engel and colleagues, revealing sEGFP, as a marker of P2X7 protein, in hippocampal dentate granule neurons and to a lesser extent CA1 pyramidal neurons after prolonged seizure (status epilepticus) [211]. These observations were confirmed in a subsequent study along with increased sEGFP amounts in microglia [212]. Likewise, neocortex neuronal cells displayed increased sEGFP amounts after prolonged seizure [213]. The sEGFP reporter mouse also helped to show that tissue-non-specific alkaline phosphatase deficiency, which causes a bone disorder and seizures, resulted in reduced sEGFP amounts in dentate gyrus neurons [214]. Increased sEGFP in astrocytes, but not microglia, has been reported in the brains of these mice during ischemic tolerance [215]. Amounts of sEGFP were increased in microglia during neuroinflammation induced by amyloid-β peptide or lipopolysaccharide [216]. This reporter mouse was also used to further investigate the role of the specificity protein factor Sp1 as a mediator of P2X7 synthesis in the brain [217]. In this study, high amounts of sEGFP were shown in peritoneal macrophages and the spleen and colocalized with Sp1 to some extent in the cortex and more clearly in the pons. Finally, this reporter mouse has been used to extensively map the distribution of P2X7 in the embryonic (E18.5) mouse brain [218]. However, a second transgenic P2X7 mouse, Tg(RP24-114E20P2X7-StrepHis-EGFP)Ani, has questioned this predominately neuronal localization of P2X7 in the brain [71]. In this mouse line, a P2X7-enhanced green fluorescent protein (EGFP) fusion protein is overexpressed under the control of a BAC-derived *P2rx7* gene promotor [71]. This model is named P2X7-EGFP reporter mouse hereafter. The functionality of the construct was confirmed in a so-called rescue mouse, in which P2X7-EGFP was crossed into the *P2rx7* KO mouse. Comparison of the EGFP signal with endogenous P2X7 in wild type (stained with a P2X7-specific nanobody) revealed identical distribution patterns. Further detailed analysis of brains from these mice identified P2X7-EGFP in microglia, Bergmann glia and oligodendrocytes but not in neurons. Likewise, an investigation of P2X7-EGFP localization in the myenteric plexus of the distal colon revealed its presence in macrophages and enteric glia but not in neurons [72]. Notably, the P2X7-EGFP overexpression per se did not result in any overt pathology or behavioral changes in these studies under normal conditions (in the absence of induced diseases). Following prolonged seizures, P2X7-EGFP was also mainly observed in microglia and oligodendrocytes but not neurons or astrocytes [219], and mice displayed increased P2X7 amounts in peripheral blood monocytes [68]. Interestingly, P2X7-EGFP reporter mice display increased susceptibility to phencyclidine-induced schizophrenia [220], decreased responsiveness to antiepileptic drugs [221] and greater stroke size after temporary middle cerebral artery occlusion [74].

Direct comparisons of the sEGFP and P2X7-EGFP mice by in situ hybridization, immunohistochemistry and flow cytometric analysis confirmed different cellular EGFP distribution, with clear neuronal localization in sEGFP mice, and localization in microglia and oligodendrocytes in P2X7-EGFP mice. In addition to microglia, levels of sEGFP were variable or absent in other cell types for which the presence of P2X7 is well described, such as macrophages, mast cells and CD4^+^ T cells [222]. The distribution of sEGFP and P2X7-EGFP was, however, comparable in satellite glial cells and Schwann cells in the spiral ganglion of the cochlea [223]. As for reasons for the aberrant cellular distribution of the sEGFP reporter, the disruption of important regulatory elements was discussed [222]. Furthermore, it was shown in this study that the sEGFP mouse also overexpresses BAC-derived P2X7 and (unlike the P2X7-EGFP mouse) P2X4.

### 3.5. Humanized Mouse Models

The term humanized mice refers to either mice expressing specific human gene products (often in place of the murine ortholog) or the engraftment of human cells into (typically) immune compromised mice [224]. In the case of the former, two humanized P2X7 mouse lines, termed P2rx7*^tm1.1(P2RX7)Jde^* and P2rx7^tm2.1(P2RX7*)Jde^, have been described [225]. In these lines, exon 2 of the *P2rx7* gene was replaced by the human *P2RX7* complementary DNA encoding exon 2–13 to express a mostly (except for exon 1) human P2X7 under the control of the murine *P2rx7* promoter [226]. The P2rx7*^tm1.1(P2RX7)Jde^* mouse was defined as a wild-type strain, while the P2rx7^tm2.1(P2RX7*)Jde^ mouse encoded the human Gln460Arg SNP [227], which has been associated with depression and anxiety in people [228,229]. Comparison of the two homozygous mouse lines and the heterozygous combination revealed disturbed sleep profiles for mice that are heterozygous for the Gln460Arg SNP [227], suggesting an increased risk for mood disorders in people heterozygous for this SNP [230]. Collectively, these studies provide the conceptual framework for developing new humanized mice lines to explore other human *P2RX7* SNPs, some of which have been associated with conditions such as inflammatory and bone disorders, infectious disease and cancer [231,232]. Moreover, flanking of the inserted sequence by loxP sites into the P2rx7*^tm1.1(P2RX7)Jde^* mouse allowed the constitutive and conditional P2X7 deletion when crossed to Cre-expressing mouse lines [226]. These whole-body knockout mice lacked any functional escape variants, while investigations of cell-type specific *P2rx7* KO mice revealed *P2rx7* mRNA expression in glutamatergic pyramidal neurons of the hippocampus as well as astrocytes, oligodendrocytes and microglia of the cortex, hippocampus, and cerebellum [226].

Humanized mice that are generated by the engraftment of human cells into (typically) immune compromised mice (xenograft models) have mainly been used to study P2X7 in various cancer types including leukemia as well as in GVHD, as discussed further below. In relation to cancer, these models typically involve the engraftment of human cancer cells into immune compromised mice [233]. Such models have afforded the opportunity to study P2X7, including P2X7 variants, in P2X7-transfected HEK-293 [233] or leukemia [234,235] cells. The investigation of xenograft models, in conjunction with ATP or BzATP and/or P2X7 antagonists, has revealed a role for P2X7 in the proliferation of colorectal [236,237] and pancreatic [238,239] cancer cells and in the invasion and migration of colorectal cancer cells [237]. Some xenograft models have revealed that P2X7 can promote the progression of acute myeloid leukemia [235,240] while mediating apoptosis and preventing disease progression in other studies [234,241]. Differences in the role of P2X7 in leukemia may be explained by the relative proportions of the pro-apoptotic and pro-proliferative P2X7A and P2X7B isoforms, respectively [240]. Likewise, there is evidence that P2X7 activation induces cell death and prevents the growth of human breast [242,243] and urinary bladder urothelial [244] cancer cells in mice. Combined, these studies highlight the complex role of tumor-derived P2X7 in cancer in addition to the anti-tumor roles of P2X7 on immune cells [245].

In relation to GVHD, the injection of human peripheral blood mononuclear cells (PBMCs) into non-irradiated NOD.Cg-*Prkdc^scid^IL2rg^tm1Wjl^* (NSG) mice, hereafter named Hu-PBMC-NSG mice, results in the development of lethal GVHD within 10 weeks [246]. Studies using this xenograft model have revealed that *P2rx7* mRNA expression is increased during GVHD [247] and that its blockade with BBG can reduce histological and clinical signs of disease and increase human regulatory T cells [129], thus confirming a role for P2X7 in this disease [248]. This model also afforded the opportunity to engraft NSG mice with PBMCs from healthy donors encoding either loss- or gain-of-function *P2RX7* SNPs [249], but the comparison of a small number of donors and genotypes did not reveal any difference between the two groups, suggesting that the donor *P2RX7* genotype does not influence GVHD development. These findings are consistent with observations in people following an allogenic hemopoietic stem cell transplantation [250]. Nevertheless, Hu-PBMC-NSG mice provide new opportunities to study other human P2X7-mediated immune cell responses in vivo, including short-term experiments prior to the development of GVHD.

## 4. Rats

### 4.1. Preclinical Rat Models

Rats (*Rattus norvegicus*) have been used as preclinical models to investigate P2X7 although less frequently than mice [54]. As for mouse models, it is beyond the scope of this article to review each study published to date. Again, some salient observations are listed below.

All P2X subunits were originally cloned from rats [81], and the use of rat models greatly advanced early P2X7 studies. In these studies, the pharmacokinetic profiles of the specific P2X7 antagonists A-740003 or A-438079 were evaluated in detail and reported to reduce neuropathic and inflammatory pain in different experimental models [251,252]. Together, these studies widened the P2X7 field to the pharmacokinetic profiling and clinical evaluation of new P2X7 antagonists in rats in an increasing number of studies. Likewise, the pharmacokinetic profiling and clinical evaluation of CE-224,535 (Section 2.3), JNJ-42253432 [253], JNJ-54166060 [254] and Lu AF27139 [255] was undertaken in rats. Thus, given such detailed characterization, rats may offer a better basis from which to design future preclinical studies of P2X7. However, these studies can be limited by the larger size of rats and corresponding increased expenses compared to mice and by the notion that some, perhaps most, of these well-characterized compounds may not be readily available to all researchers due to their clinical potential and testing in people. More recent studies using the local delivery of small interfering RNA to silence P2X7 in rats have revealed a role for this receptor in diabetic neuropathy [256,257,258], epilepsy [259] and intracerebral hemorrhage [260]. This provides alternative therapies to the use of small molecule inhibitors to investigate P2X7 in vivo. Of note, acute stress (1 h immobilization) can potentially lead to ATP release and the subsequent activation of the P2X7-inflammsome pathway to induce IL-1β release in rats [261]. Although this study was part of a larger study examining the role of P2X7 in psychological stress, it highlights possible confounding effects of extracellular ATP and P2X7 in animal models where restraint procedures are employed.

### 4.2. Polymorphic P2X7 Variants in Rats

Similar to mice, it should be noted when considering rats as models of P2X7 that the amount or activity of this receptor may also vary between or even within rat strains. For example, both *P2rx7* mRNA expression and amounts of P2X7 are greater in macrophages from Wistar Kyota (WKY/NCrl) rats compared to those from Lewis (LEW/CRL) rats [262]. The exact cause of this difference remains unknown, but gene sequencing revealed that this difference is not due to a non-synonymous mutation but is likely due to deletions or insertions in the promotor region of the *P2rx7* gene or elsewhere in the genome [262]. In another example, comparing Sprague–Dawley *Uox* (uricase) knockout rats with acute gouty arthritis to those without this disease identified a mutated locus (1016) in the 5′ untranslated region of that *P2rx7* gene that was associated with gout development [263]. This infers that *P2rx7* gene variants exist between rats of the same background, further complicating rat investigations, at least in this rat line. Although further studies are required to substantiate these findings, researchers should consider potential differences in rat P2X7 in future studies.

### 4.3. P2rx7 Gene Knockout Rat Models

In recent years, several *P2rx7* KO rat lines have become available (Table 4). CRISPR/Cas9 has been used to globally delete the *P2rx7 gene* in PCK/CrljCrl-*Pkhd1pck*/Crl (PCK) rats, derived from Sprague–Dawley rats encoding a mutation in the Pck locus and serving as a model of inherited polycystic kidney disease [163]. Rats with this deletion, compared to wild-type PCK rats, had impaired renal cyst development and ATP urinary release, with the latter corresponding to decreased amounts of renal pannexin-1 protein. Zinc finger nuclease technology has been used to globally delete the *P2rx7 gene* in Wistar Kyota rats, although the presence of escape variants in the brain could not be excluded [164]. This deletion did not protect rats from nephrotoxic nephritis, glomerulonephritis, or autoimmune nephritis, indicating P2X7 is not essential for the development of these disorders. Of note, A-438079 protected both *P2rx7* KO and wild-type rats from nephrotoxic nephritis, indicating off-target effects of this P2X7 antagonist and highlighting the potential utility of *P2rx7* KO animals to study the specificity of P2X7 antagonists. Finally, a published conference abstract has reported the use of CRISPR/Cas9 to globally delete the *P2rx7 gene* in F344/OciCrl rats, resulting in impaired endothelial-dependent vasodilation in male, but not female, rats [165].

## 5. Guinea Pigs

Despite the cloning of P2X7 from the guinea pig (*Cavia porcellus*) [99] and early studies reporting P2X7 protein and functional expression in the enteric nervous system of the small intestine [264] and P2X7 protein in smooth muscle of the *vas deferens* and epithelium of the urinary bladder [265] of guinea pigs, this species has been used infrequently to study P2X7. More recent studies of guinea pigs have reported functional P2X7 in intestinal myenteric neurons [266], *P2rx7* mRNA expression in the urinary bladder [267] and the up-regulation of P2X7 in peripheral blood leukocytes in an allergen (ovalbumin) sensitization model [268]. The main site in which P2X7 has been studied in the guinea pig is the cochlear, with low amounts of P2X7 protein reported in the outer hair cells of the cochlear [269,270] but little change in amounts of this protein in response to noise [271]. Another study has dismissed a role for P2X7 in auditory neurotransmission in guinea pigs [272]; however, P2X7 activity in this study was investigated using BzATP only, which was subsequently shown not to activate recombinant guinea pig P2X7 [99]. Based on studies in other species, P2X7 may contribute to glial-mediated inflammatory processes and contribute to auditory neuropathy and hearing loss under some conditions [273].

## 6. Rabbits

Like guinea pigs, P2X7 was investigated in rabbits (*Oryctolagus cuniculus domesticus*) in a small number of early studies with the earliest of these reporting minimal amounts of P2X7 protein in the endothelium of the aorta of normal rabbits with no changes in amounts following balloon injury in vivo [274]. Another early study reported the presence of functional P2X7-like responses in rabbit airway ciliated cells, but this study was unable to confirm the presence of this protein in these cells [275]. P2X7 activation has also been reported to mediate Ca^2+^ fluxes [276] or stimulate NF-κB [277] in rabbit osteoclasts, and prostaglandin E_2_ release and cell death in rabbit articular chondrocytes [278], but the role of this receptor in the bone physiology and pathophysiology in rabbits in vivo is yet to be established in contrast to mice and human bone disorders [4]. P2X7, as well as P2X1–P2X5, protein is increased in ischemic bladders in rabbits [279].

Other studies have suggested a role for P2X7 activity in rabbits in vivo. The purported P2X7 antagonist Brilliant blue FCF can reduce intimal thickening following vein engraftment in rabbits [280], suggesting a role for P2X7 activation in this process. However, others have shown that this compound impairs mouse pannexin-1, but unlike BBG [110], not human P2X7 [281], questioning the earlier results in rabbits. This also highlights the importance of studies on recombinant receptors to determine the species-specific pharmacological properties for the interpretation of data from in vitro and in vivo studies. The P2X7 protein has been observed in the retinas of healthy rabbits, with this protein extending to the outer layer and some small blood vessels in the retinas of diabetic rabbits [282]. In this study, BzATP preferentially reduced retinal blood velocity in diabetic compared to healthy rabbits, which is a process that was blocked by the P2X7 antagonist oxidized ATP [283]. Further studies relating to the eye have reported that P2X7 activation mediates benzalkonium chloride (eye drop preservative)-induced cytotoxicity [284] and sodium dodecyl sodium-induced cytotoxicity [285] in rabbit and human corneal cells. Furthermore, the latter studied revealed that high molecular weight hyaluronan could prevent this process in vitro in human corneal cells and in vivo in rabbits, but direct evidence for the action of P2X7 in this process in vivo was not investigated.

## 7. Monkeys

Despite the increasing use of nonhuman primates to study P2X7 antagonists and radiolabeled ligands in preclinical studies (see Section 2.3), few studies have reported the study of P2X7 directly in these animals. The P2X7 protein has been observed in the inner nuclear, inner plexiform and ganglion cell layers, but not glia, of the retinas from rhesus macaques (*Macaca mulatta*) [286]. Meanwhile, *P2rx7* mRNA expression has been reported in bone-marrow derived macrophages and microglia from these animals [287]. Along with studies of recombinant rhesus macaque P2X7 [96], evidence of functional P2X7 in rhesus macaques has been demonstrated by studies of IL-1β release, with ATP-induced release of this cytokine from bone-marrow derived macrophages and microglia fully and partly mediated by P2X7 activation, respectively [287].

## 8. Dogs

Following mice and rats, dogs (*Canis lupus familiaris*) are arguably the best studied animal regarding P2X7. The presence of P2X7 in dogs has recently been extensively reviewed [288]. In addition to detailing recombinant P2X7 and polymorphic variants of canine P2X7, this article provides a comprehensive review of the molecular and functional expression of P2X7 in cells and tissues from dogs, with P2X7 reported in erythrocytes, T and B cells, monocytes, and macrophages as well as adipose stem cells, kidney cells, nervous system cells including brain cancers, and possibly myenteric plexus neurons. Moreover, this article provides a comprehensive overview of the published studies reporting the pharmacokinetics and safety of P2X7 antagonists in dogs. Apart from these preclinical studies, however, the study of P2X7 in dogs in vivo has not been reported.

## 9. Cats

Like guinea pigs, rabbits and rhesus macaques, a small number of studies have reported the presence of P2X7 in cats (*Felis catus*). The P2X7 protein is found in endothelium and muscle cells, but not subepithelial neurons, of the urinary bladder of cats [289]. In contrast, the P2X7 protein has been reported in the neurons of urinary bladder intramural ganglia [290] and the upper sacral dorsal root ganglia [291] from cats. However, these three studies, from the same group, used the same anti-P2X7 antibody (Roche, Palo Alto, CA, USA); thus, further studies are required to confirm the presence of P2X7, including functional receptors, in the cat. Of note, using an anti-P2X7 antibody, which recognizes nonfunctional P2X7 [292], the presence of such receptors has been identified in a nonresectable nasal squamous cell carcinoma in a cat, and furthermore, treatment with this antibody resulted in the complete resolution of this lesion [293]. An equivalent antibody (BIL010t) was subsequently tested in a phase 1 clinical trial in human basal cell carcinoma, with partial responses seen in 65% of patients [294]. However, further clinical testing of this biologic has not been reported, despite the development of a fully humanized single-chain antibody (BIL03s) and a murine mAb (BPM09) [295].

## 10. Zebrafish

Zebrafish (*Danio rerio*) are a common experimental model used in many contexts including purinergic signaling [296], with several studies reporting the use of these animals to study P2X7 in vivo (Table 5). One possible caveat in the use of zebrafish as a model to study this receptor is that zebrafish P2X3 shows an agonist profile like that of mammalian P2X7 [297]. Direct comparisons of agonist and antagonist profiles of zebrafish P2X7 and P2X3 may help address this potential limitation.

To date, the most common studies of P2X7 in zebrafish have been xenograft models of human breast cancer. Using MDA-MB-435s cells, which expressed *P2rx7* mRNA, and the P2X7 antagonist, A-438079, the first of these studies established a role for P2X7 in cancer invasiveness, as a model of metastasis [298]. In contrast, the invasiveness of human esophagus cancer EO33 cells, which did not express *P2rx7* mRNA, was not impaired by A-438079 in this study. This group subsequently confirmed a role for P2X7 in this process, with the novel P2X7 antagonist, anthraquinone emodin, impairing the invasiveness of P2X7 positive MDA-MB-435s cells but not P2X7 negative human breast cancer MDA-MB-468 cells [299]. This study highlights the use of zebrafish as an additional in vivo model in the establishment and testing of new P2X7 antagonists. To this end, others used zebrafish to demonstrate the ability of four novel 1-piperidinylmidazole-based P2X7 antagonists to impair the invasiveness of human breast cancer MDA-MB-231 cells [44].

Other studies have used zebrafish and P2X7 antagonists or *p2rx7* gene knockdown techniques to establish a role for P2X7 in disorders such as polycystic kidney disease [300], seizure [301], and inflammation during tissue injury [302]. In contrast, the use of A-740003 was unable to establish a role for P2X7 activation in CuS0_4_-induced inflammation in this species, despite probenecid impairing this process and indicating a potential role for pannexin-1 in this inflammation model [303]. Likewise, the use of these same two compounds revealed a role for pannexin-1 but not P2X7 in a model of pain in zebrafish [304]. Another study reported that the reduction in hepatic stasis by the plant flavanol, quercetin, was associated with decreased *P2rx7* mRNA expression, indirectly implicating a role for P2X7 in this disorder [305]. Zebrafish have also been used to assess the role of P2X7 in toxicity. Heavy metal (HgCl_2_) toxicity decreased *p2rx7* mRNA expression in zebrafish, while the co-administration of HgCl_2_ and ATP, but not each compound alone, reduced survival, with effect of the combined treatment prevented by A-740003 [306].

**Table 5:** Study of P2X7 in zebrafish models.

Disease	Key Findings ^1^	Reference
Metastasis (MDA-MB-435s, EO33)	A-438079 ↓ MDA-MB-435s but not EO33 cell invasion	[298]
Metastasis (MDA-MB-435s, MDA-MB-468)	Emodin ↓ MDA-MB-435s but not MDA-MB-468 cell invasion	[299]
Metastasis (MDA-MB-231)	P2X7 antagonists ↓ cell invasion	[44]
Polycystic kidney disease (*pkd2* morphant)	*pkd2* morphant ↑ *p2rx7* expression, OxATP, A438079 and *p2rx7* knockdown ↓ cyst formation	[300]
Seizure (pentylenetetrazol-induced)	Probenecid and A-438079 ↓ seizure activity	[301]
Tissue injury (tail transection)	KN-62 and BBG ↓ neutrophil and macrophage recruitment, and *il1b* mRNA expression	[302]
Inflammation (CuS0_4_-induced)	Probenecid but not A-740003 ↓ inflammation	[303]
Pain (acetic acid-induced)	Probenecid but not A-740003 ↓ pain	[304]
Hepatic steatosis (ethanol-induced)	Quercetin ↓ *p2rx7* expression	[305]
Heavy metal toxicity (HgCl_2_-induced)	HgCl ↓ *P2rx7* expression, HgCl+ATP ↓ survival (A740003 ↑ survival)	[306]
Retinal degeneration (CoCl_2_-induced)	BzATP ↑ degeneration (↓ by A-740003), CoCl_2_ ↑ *p2rx7* expression, A-740003 ↑ CoCl_2_-induced degeneration	[307]
*Mycobacterium marium* infection	Clemastine ↓ mycobacterium growth	[308]

^1^ Abbreviations: ↓, decreased; ↑, increased; ATP, adenosine 5’-triphosphate; BBG, Brilliant Blue G; BzATP, 2′(3′)-O-(4-benzoylbenzoyl) ATP; OxATP, oxidized ATP.

In contrast to the role of P2X7 in promoting various disorders, autocrine P2X7 activation has been shown to have a protective role in a zebrafish model of retinal degeneration, with A-740003 worsening CoCl_2_-induced phototoxicity [307]. This worsened retinal degeneration was associated with increased Muller cell proliferation and microglia activation. In this same study, the injection of BzATP into non-injured retinas also induced photoreceptor damage and Muller cell proliferation, which could be prevented by the co-injection of A-740003, revealing opposing roles for P2X7 on phototoxicity.

Zebrafish can also be used in the screening of compound libraries to identify new P2X7 modulators. Based on the established role of P2X7 in tuberculosis in people [309], 1200 approved drugs were screened in a zebrafish model of tuberculosis, leading to the identification of the clinically approved anti-histamine clemastine as a positive P2X7 modulator [308]. Although an earlier in vitro drug library screen had identified this compound as a positive modulator of human and mouse P2X7 [310], this later study established the use of zebrafish to screen for P2X7 modulators [308]. Moreover, this study established the use of *p2rx7* knockout and other transgenic zebrafish to establish that the anti-mycobacterial activity of clemastine was due to P2X7 activity on macrophages. Consistent with the presence of P2X7 on zebrafish macrophages, isolated zebrafish coelomic cells, which include representatives of peripheral blood cells, macrophages and bone-marrow derived cells, possess functional receptors as assessed by ATP-induced dye uptake, which can be blocked by either oxidized ATP, KN-62 or BBG [311].

## 11. Other Fish Species

Further to the use of zebrafish, P2X7 has been investigated in other fish species. Initially, P2X7 was cloned and studied from seabream, where it was shown to induce cell death but not IL-1β maturation or release in leukocytes [312]. Subsequently, it was shown that P2X7 activation was unable to stimulate capsase-1 activity in these cells [313]. In contrast, BzATP or ATP, respectively, was reported to induce caspase-1 activity [313], phosphatidylserine exposure, microvesicle shedding, and IL-1β maturation and release [312] in the seabream fibroblast cell line SAF-1.

*p2rx7* mRNA is present in macrophages as well as the spleen, head kidney, gill, liver, muscle, intestine, and heart from ayu sweetfish (ayu) (*Plecoglossus altivelis*) [314]. This study also reported that the P2X7 protein is present in ayu macrophages and that the activation of this receptor in these cells can induce cell death as well as phagocytosis and killing of the bacterium *Vibrio anguillarum*. Furthermore, this study determined the complementary DNA sequence of ayu P2X7, predicting a protein 574 amino acid resides in length, and used this sequence to generate a recombinant ectodomain fragment, which was then used to generate an antibody (aEPAb) capable of detecting P2X7 protein and blocking P2X7 activity in macrophages. A subsequent study using ayu macrophages showed that the endogenous antimicrobial peptide, cathelicidin, induced chemotaxis, the synthesis of mRNA transcripts coding for IL-1β, IL-10 and tumor necrosis factor, oxidative bursts, phagocytosis, and *V. anguillarum* killing in a P2X7-dependent manner [315], supporting interactions between antimicrobial peptides and P2X7 observed in mice and humans in other infection types [316].

Like the findings with ayu, another study has reported the cloning of P2X7 from the rainbow trout (*Oncorhynchus mykiss*), predicting a protein 551 amino acid resides in length and with *p2rx7* mRNA transcripts detected in B cells from this fish [317]. This study also revealed that the cathelicidins, CATH-1a and CATH-2a, could induce reactive oxygen species formation and promoted *Escherichia coli* killing in trout lymphocytes, with the authors postulating a role for P2X7 in this process.

A study of the Japanese flounder (*Paralichthys olivaceus*) reported high *p2rx7* mRNA expression in the hepatopancreas, with intermediate expression in the blood, brain, gonad, heart, intestine, muscle and skin, and limited expression in the gill, head and trunk kidneys, and spleen [101]. In addition to producing a functional recombinant receptor, this study and another [318] reported that P2X7 activation could promote the synthesis of mRNA transcripts coding for IL-1β and IL-6 in Japanese flounder macrophages. Furthermore, ATP induced the mRNA transcript coding for caspase-1 and caspase-1 activity in macrophages and lymphocytes from this species [319], but direct evidence for P2X7 in these roles was lacking. Likewise, this same approach revealed that ATP can induce mRNA transcripts coding for caspase-2, -3, -6 and -8 and the activity of these caspases in these same cell types [320]. Notably, CD39 can limit ATP-induced responses in Japanese flounder [321], which is in keeping with well-known observations in rodents and humans [322]. Extracellular ATP has also been shown to cause other immunomodulatory activities; cytokine gene transcription as well as reactive nitrogen and oxygen species production have been reported in Japanese flounder cells, but again, direct evidence for P2X7 in each of these responses was lacking [323]. Finally, co-transfection studies have revealed that apoptosis-related serine/threonine kinase 17A increases the P2X7-mediated apoptosis of Japanese flounder FG-9307 cells [324].

## 12. Conclusions

Studies of different species of animals, particularly mice and rats, have greatly advanced the investigation of P2X7, which has helped establish numerous physiological and pathophysiological roles of this receptor in human health and disease. The use of animals has contributed to the generation of antibodies, nanobodies, recombinant receptors and radioligands to study P2X7, which have characterized the pharmacology profiles, revealed the cellular and tissue distribution, and determined the functional roles of this receptor. The use of several species has also contributed to the pharmacokinetic testing of P2X7 antagonists, which has led to clinical trials of drugs targeting P2X7 in humans. The development of transgenic mouse models, including *P2rx7* KO and reporter mice, humanized P2X7 mice, as well as *P2rx7* KO rats provide innovative tools, which will continue to help in the exploration of this receptor. Meanwhile, the limited number of studies in guinea pigs, rabbits, rhesus macaques, dogs, cats, zebrafish, and other fish species have provided opportunities to further define the roles of P2X7 in health and disease. Ultimately, the study of P2X7 will result in new knowledge that can be applied to improve the health and well-being of humans and other animal species alike.

## Figures and Tables

**Table 1:** P2X7 agonists and modulators of P2X7 activity specifically referred to in this article.

	Compound or Biologic ^1^
Agonists	ATP, BzATP, NAD^+^
Partial agonists	Adenosine-5′-O-(3-thio) triphosphate, 2-methylthio-ATP
Positive modulators	Clemastine, HEI3090, anti-murine nanobody (14D5)
Non-selective antagonists	BB FCF, BBG, emodin, KN-62, OxATP, PPADS, probenecid
Selective antagonists	A-438079, A-740003, AZ101606120, CE-224,535, JNJ-42253432, JNJ-47965567, JNJ-54166060, Lu AF27139, 1-piperidinylmidazole-based antagonists ^2^
Inhibitory antibodies	Anti-ayu P2X7 polyclonal antibody (aEPAb), anti-human P2X7 mAb (clone L4), anti-murine P2X7 mAb (clone 1F11)
Inhibitory nanobodies	Anti-human nanobody (Dano1), anti-murine nanobody (13A7)

^1^ Abbreviations: ATP, adenosine 5′-triphosphate; BB FCF, Brilliant Blue FCF; BBG, Brilliant Blue G; BzATP, 2′(3′)-O-(4-benzoylbenzoyl) ATP; mAb, monoclonal antibody; NAD^+^, nicotinamide adenosine dinucleotide; OxATP, oxidized ATP; PPADS, pyridoxalphosphate-6-axophenyl-2′-4′-disulfonic acid. ^2^ 1-Piperidinylmidazole-based compounds 12g, 13k, 17d and 20b [44].

**Table 4:** Conventional *P2rx7* gene knockout mouse and rat models.

Species	Technology	Target Site (Defect)	Reference
Mouse	LacZ-neomycin cassette ^1^	Exon 1 (deletion)	[153]
Mouse	Neomycin cassette	Exon 13 (truncation)	[156]
Mouse	LacZ-neomycin cassette	Exon 2 and 3 (substitution)	[158]
Mouse	Short hairpin cassette	Exon 3 (knockdown)	[161]
Mouse	CRISPR/Cas9	Exon 2 (deletion)	[159]
Rat	CRISPR/Cas9	Exon 2 (frameshift mutation)	[163]
Rat	ZFN ^2^	Exon 10 (2 bp insertion)	[164]
Rat	CRISPR/Cas9	Exon 2 (stop codon)	[165]

^1^ Full details of the generation of this *P2rx7* gene knockout mouse were reported in two subsequent publications [154,155]. ^2^ Abbreviations: bp, base pair; ZFN, zinc finger nuclease.

## Data Availability

Not applicable.
